# Maternal and neonatal outcome in pregnant women undergone induction of labor at Muhimbili National Hospital, Dar Es Salaam, Tanzania

**DOI:** 10.1186/s12884-024-06578-w

**Published:** 2024-05-24

**Authors:** Shweta Jaiswal, Willbroad Kyejo, Charles Kilewo

**Affiliations:** 1 Department of Obstetrics and Gynecology, Aga Khan University Medical College, Dar Es Salaam, Tanzania; 2 Department of Family Medicine, Aga Khan University Medical College, Dar Es Salaam, Tanzania; 3 Department of Obstetrics and Gynecology, Muhimbili University of health and Allied Science, Dar Es Salaam, Tanzania

**Keywords:** Labor induction, Maternal outcomes, Neonatal outcomes, Retrospective study

## Abstract

**Introduction:**

Labor induction is a common obstetric intervention aimed at initiating labor when spontaneous onset is delayed or deemed necessary for maternal or fetal well-being. Despite its widespread use, the practice’s impact on maternal and neonatal outcomes remains a subject of ongoing research and debate. This study aims to evaluate the maternal and neonatal outcomes associated with labor induction in a tertiary hospital setting in Tanzania.

**Methodology:**

A descriptive analytical cross-sectional study was conducted over a seven-month period from January 2021 to July 2021 at Muhimbili National Hospital in Dar es Salaam, Tanzania. A total of 120 pregnant women who underwent labor induction during this period were included in the analysis. Data on maternal demographics, obstetric characteristics, indications for induction, methods of induction, labor outcomes, and neonatal outcomes were collected from medical records and analyzed descriptively.

**Results:**

Among 4773 deliveries during the study period, 120 women underwent labor induction, accounting for 120 (2.5%) of all deliveries. The most common indications for induction were postdate pregnancy 60 (50%), hypertensive disorders of pregnancy 38 (31.7%), and premature rupture of membranes 22 (17.5%). The majority of induced women 74 (61.7%) delivered vaginally, with 46 (38.3%) undergoing cesarean section. Maternal complications were minimal, with the most common being failed induction of labor 17 (14.2%). Neonatal outcomes were generally positive, with 120 (100%) of neonates having Apgar scores of 7 or higher at five minutes, although 10 (8.3%) required admission to the neonatal ward for further care.

**Conclusion:**

Labor induction at Muhimbili National Hospital demonstrated favorable maternal and neonatal outcomes, with low rates of maternal complications and positive neonatal Apgar scores. Postdate pregnancy emerged as the most common indication for induction. While the study highlights the benefits of labor induction, its retrospective nature and single-center setting limit the generalizability of findings. Prospective studies with larger sample sizes are warranted to validate these findings and inform evidence-based obstetric practices.

**Supplementary Information:**

The online version contains supplementary material available at 10.1186/s12884-024-06578-w.

## Background

Induction of labor (IOL) is a common obstetric intervention aimed at initiating uterine contractions artificially before spontaneous onset, typically at a viable gestational age, with the goal of achieving vaginal delivery. It encompasses both pregnant women with intact membranes and those with spontaneous rupture of membranes who have not yet entered labor [1]. The procedure is performed for various reasons, including medical, obstetric, or maternal-fetal indications.

Successful vaginal delivery following IOL is influenced by several factors, including multiparity, favorable cervical status, and gestational age [2]. The Bishop score, a scoring system based on cervical examination, is commonly used to predict the likelihood of vaginal delivery. Factors such as cervical dilatation, effacement, consistency/ripening, station of the baby’s head, and position of the cervix are assessed to determine the readiness for induction [3,4].

The history of labor induction dates back centuries, with methods evolving from mechanical techniques to pharmacological interventions. Mechanical methods involve the use of catheters and dilators to exert pressure on the cervix, while pharmacological methods utilize agents like prostaglandins (PGE1 and PGE2) and oxytocin to ripen the cervix and stimulate uterine contractions [5, 6].

Prostaglandins, including misoprostol (PGE1 analogue) and dinoprostone (PGE2), are commonly used for cervical ripening and induction of labor. Misoprostol can be administered orally, vaginally, rectally, or intracervically, while dinoprostone is available as an intravaginal insert or intracervical gel [7].

Oxytocin, an octapeptide hormone, stimulates myometrial contractions and is administered intravenously to induce or augment labor. It is titrated to achieve optimal contraction patterns while monitoring for adverse effects like uterine hyperstimulation and hypotension [6, 8].

Induction of labor rates vary globally, with higher rates observed in developed countries compared to developing regions. Indications for IOL include hypertensive disorders of pregnancy, post-term gestation, premature rupture of membranes (PROM), and fetal/maternal complications. IOL has been associated with reduced cesarean section rates and improved maternal and neonatal outcomes [9].

Despite its benefits, there are challenges associated with IOL, including failed inductions, maternal complications (e.g., uterine hyperstimulation, postpartum hemorrhage), and neonatal concerns (e.g., low Apgar scores, NICU admissions). Understanding the indications, outcomes, and complications of IOL is essential for optimizing obstetric care and reducing adverse events.

In Tanzania, a low-resource setting with limited healthcare resources, the rate of cesarean sections is rising, particularly in tertiary referral hospitals like Muhimbili National Hospital (MNH). However, the prevalence and outcomes of IOL at MNH remain unclear. Investigating the maternal and neonatal outcomes of IOL in this setting is crucial for informing clinical practice, improving patient care, and addressing the rising rates of cesarean deliveries.

## Methodology

The research conducted at Muhimbili National Hospital (MNH) in Dar es Salaam, Tanzania, utilized a descriptive analytical cross-sectional design spanning a duration of six months, from January to July 2021. Muhimbili National Hospital (MNH) is a tertiary referral and teaching hospital located in Dar es Salaam, Tanzania. It serves as a pivotal healthcare institution catering to the healthcare needs of the city’s population and those from surrounding regions. MNH is affiliated with Muhimbili University of Health and Allied Sciences (MUHAS), further enhancing its role as a center for medical education, research, and healthcare delivery.

The hospital provides comprehensive medical services across various specialties, including obstetrics and gynecology. With its status as a tertiary referral center, MNH receives complex cases and referrals from lower-level healthcare facilities, including health centers and peripheral hospitals, both within Dar es Salaam and from other regions of Tanzania.

Annually, MNH handles a substantial number of deliveries, contributing significantly to maternal and neonatal healthcare in Tanzania. The exact number of annual births at MNH varies, but it typically encompasses about 10,870 to 11,000 deliveries. This high volume of deliveries underscores the hospital’s role as a major center for obstetric care in the region.

In recent years, there has been a notable increase in the cesarean section (CS) rate at MNH, mirroring global trends in rising CS rates. The CS rate at MNH has been around 40%, indicating a significant proportion of deliveries are conducted via cesarean section. This trend may reflect various factors, including changes in obstetric practices, increased demand for cesarean deliveries, and clinical indications for surgical intervention during childbirth.

Despite efforts to improve maternal and neonatal healthcare, MNH continues to grapple with challenges related to adverse perinatal and maternal outcomes. Adverse outcomes encompass a range of complications and adverse events, including maternal morbidity and mortality, stillbirths, neonatal deaths, and complications arising from childbirth. These adverse outcomes may be influenced by various factors, including limited resources, inadequate access to healthcare, and sociodemographic determinants of health.

The primary objective of this study was to investigate and analyze the maternal and neonatal outcomes among pregnant women undergoing induction of labor at MNH.

The sample size calculation using a formula for single proportion.

n = Z^2^ x P (1- P)/ E^2^

Whereby n = Expected sample size, Z score = 1.96 for 95% confidence interval, the specific outcome used to calculate the sample size in our study was the prevalence of maternal complications associated with induction of labor within the Nigerian population, as reported in a study conducted in Niger by Ayuba II et al. [10]. The proportion in the sample size calculation represents the expected prevalence of this outcome within our study population *P* = 15%, e = Margin of error-0.05.

*n* = 1.96 ^2^ × 0.15 (1- 0.15)/ 0.05^2^

*n* = 100. Inflate of 20% will be added to the sample size accounting for spoiled records or missing data (*n* + 20).

Therefore, the minimum sample size will be **120** participants.

Employing a convenient sampling technique, participants were selected from obstetric clients delivering at MNH after 28 weeks of gestation, with exclusions made for cases involving intrauterine fetal death or congenital malformations. A structured data collection tool was utilized to capture a wide array of information, including socio-demographic characteristics, obstetric history, indications for labor induction, Bishop score assessments, induction to delivery intervals, failure rates, and subsequent maternal and neonatal outcomes. The data were sourced from various records such as delivery registers, antenatal cards, round books, and patient files.

The collected data underwent meticulous management processes, including coding and entry into SPSS version 20 for analysis. Data cleaning procedures were implemented to rectify any inconsistencies or missing variables within the dataset. Descriptive statistical methods were employed to summarize the demographic data and categorical variables, allowing for a comprehensive examination of the maternal and neonatal outcomes associated with induction of labor at MNH.

The study’s findings were presented through detailed tables, charts, and statistical summaries to facilitate a clear and nuanced understanding of the observed outcomes. These outcomes encompassed various aspects such as mode of delivery, induction to delivery intervals, intra-partum and postpartum complications for mothers, as well as indicators like Apgar scores, meconium-stained liquor, neonatal admissions, and neonatal deaths for infants.

## Results

**Fig. 1:**
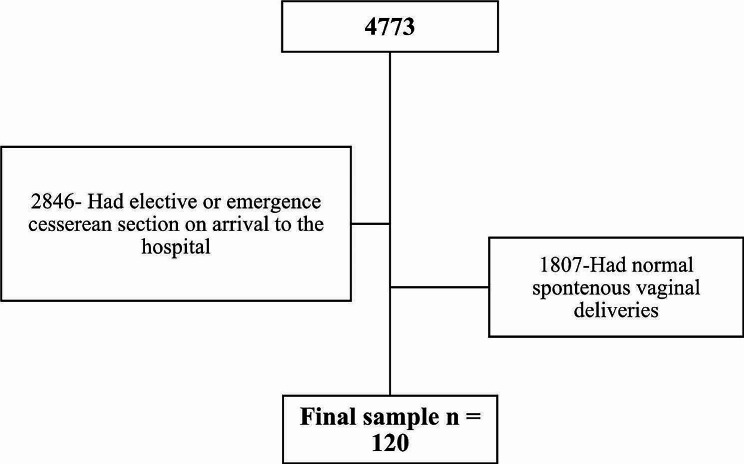
Participant recruitment flow

During the study period spanning seven months from January to July 2015, there were a total of 4773 deliveries at MNH, comprising 2846 caesarean sections (both elective and emergency) and 1927 spontaneous vaginal deliveries. Out of these, 120 pregnant women with live fetuses underwent induction of labor and were included in the outcome analysis (Fig. 1).

Table 1 presents the social demographic and obstetric characteristics of the study population at MNH in 2014. The mean age of the participants was 28.72 (SD 5.47) years, with the majority falling in the age group of 26 to 30 years. Most women attained secondary education 67 (55.8%), were married 105 (87.5%), and were multigravida 72 (60.2%).

**Table 1:** Social demographic and obstetrics characteristics of the study population at MNH 2014 (*n* = 120)

Characteristic	Category	Frequency	Percent
**Age**			
	< 20	3	2.5
	20–25	30	25.0
	26–30	38	31.7
	31–35	28	23.3
	> 35	21	17.5
**Education**			
	Primary school or less	35	29.2
	Secondary school	67	55.8
	College/University	18	15.0
**Marital status**			
	Single	5	4.2
	Married	105	87.5
	Separated/Divorced	10	8.3
**Occupation**			
	Housewife	44	36.7
	Student	3	2.5
	Petty traders	57	47.5
	Civil servants	16	13.3
**Gravidity**			
	Primigravida	47	39.2
	Multigravida	73	60.2
**Gestation age**			
	< 36	11	9.2
	36–40	49	40.8
	≥ 40	60	50.0
**Mode of Delivery**	SVD	74	62
	CS	46	38
**Bishop Score**	< 7	32	27
	7–10	88	73

Table 2 provides a frequency distribution of maternal and neonatal outcomes after induction of labor at MNH. The majority of pregnant women 74 (61.7%) had spontaneous vaginal deliveries, while 46 (38.3%) underwent emergency caesarean sections. The most common maternal complication was failed induction of labor 17 (14.2%). Nearly all neonates 119 (99.2%) had Apgar scores of 7 or above at 5 min, while 8 (6.7%) had meconium-stained liquor. Additionally, 10 (8.3%) of neonates were admitted to the neonatal ward for further management.

**Table 2:** Frequency distribution table showing maternal and neonatal outcomes after IOL at MNH, (*n* = 120)

Outcome	Frequency	Percent
**Route of delivery**		
SVD	74	61.7
Caesarean sections	46	38.3
**Maternal Complication**		
Failed induction of labour	17	14.2
PPH	3	2.5
Retained placenta	1	0.8
No complications	99	82.5
**Apgar score at 5 min**		
<7	1	0.8
≥7	119	99.2
**Meconium-Stained liquor**		
Yes	8	6.7
No	112	93.3
**Admission to neonatal ward for further management**		
Yes	10	8.3
No	110	91.7

Table 3 outlines the indications for induction of labor, with post-date pregnancy being the most common indication, followed by hypertensive disorders of pregnancy and premature rupture of membranes.

**Table 3:** Frequency distribution table showing Indications for Induction of Labour

INDICATIONS	INDUCTION OF LABOUR		TOTAL
	ELECTIVE	EMERGENCY	
Post date	54 (90%)	6 (10%)	60
**Hypertensive disorders of pregnancy**	**1 (1.7%)**	**37 (58.1%)**	**38**
**PROM**	**0 (0%)**	**21 (33.9%)**	**21**
**IUGR**	**0 (0%)**	**1 (1.6)**	**1**
**BOH**	**5 (9.7)**	**0 (0%)**	**5**

Table 4 presents the induction to delivery interval categorized by method of induction and mode of delivery. The majority of women who received misoprostol delivered in less than 12 h, while those who received vaginal dinoprostone had a significant proportion deliver between 12 and 24 h. Among those who received titrated oxytocin, most delivered in less than 12 h. The induction to delivery interval was shorter for women who had spontaneous vaginal deliveries compared to those who underwent emergency caesarean sections.

**Table 4:** Frequency distribution table showing induction to delivery interval (*n* = 120)

METHOD OF INDUCTION	Induction delivery interval			TOTAL
	< 12 h	12–24 h.	> 24 h.	
Vaginal misoprostol + oxytocin	16 (41.0%)	14 (35.9%)	9 (23.1%)	39
**Vaginal dinoprostone + oxytocin**	**21 (33.3%)**	**28 (44.4%)**	**14 (22.2%)**	**63**
**Titrated oxytocin**	**11 (64.7%)**	**6 (35.3%)**	**0 (0%)**	**17**
**Trans cervical balloon catheterization oxytocin**	**1 (100%)**	**0 (0%)**	**0 (0%)**	**1**

## Discussion

The study revealed several key findings regarding the maternal and neonatal outcomes associated with labor induction at Muhimbili National Hospital. These findings include the low rate of induction compared to developed countries, the predominant indications for induction, the mode of delivery among induced women, the incidence of maternal complications, and the neonatal outcomes following induction. Each of these findings contributes to our understanding of the efficacy and safety of labor induction in this setting and has implications for obstetric practice and patient care.

The study encompassed 120 pregnant women who underwent labor induction at the maternity labor ward within a span of six months from January to july 2021. Among the 4773 deliveries recorded during this period, induction of labor accounted for only 2.5%, a notably low figure compared to rates reported in developed countries such as the United States and the United Kingdom [11, 12], where induction rates range from 20 to 25%. This discrepancy might be attributed to varying practices, including patient and clinician preferences, as well as the availability of advanced cervical ripening agents.

The maternal outcomes observed in our study following labor induction are integral to understanding the efficacy and safety of this obstetric intervention. While the majority of women experienced favorable outcomes, it is crucial to delve deeper into the specific maternal complications encountered and their implications for clinical practice.

One of the primary maternal outcomes of interest was the mode of delivery following labor induction. Our study revealed that a significant proportion of women achieved spontaneous vertex delivery, reflecting successful progress through the stages of labor. Morever, a notable subset of women underwent cesarean sections, suggesting instances where labor progression was hindered or complications necessitated surgical intervention, the cesarean section (CS) rate among women undergoing labor induction in this study (38.3%) is below the overall CS rate of the hospital.

Among the observed maternal complications, failed induction of labor emerged as a noteworthy concern, affecting a proportion of women undergoing labor induction. Failed induction can result from various factors, including inadequate cervical ripening, uterine dysfunction, or fetal malposition.

Postpartum hemorrhage (PPH) and retained placenta were among the other maternal complications identified in our study. PPH, characterized by excessive bleeding following childbirth, can arise due to uterine atony, genital tract trauma, or coagulation disorders. Prompt recognition and management of PPH are essential to prevent adverse maternal outcomes, including maternal morbidity and mortality. Similarly, retained placenta, although less common, can lead to complications such as uterine infection or hemorrhage if not managed expediently.

While our study did not report cases of severe maternal morbidity or mortality, it is imperative to acknowledge the potential risks associated with labor induction, including uterine rupture, perineal trauma, and infection. Vigilant monitoring and timely intervention are paramount to mitigate these risks and ensure optimal maternal outcomes.

Comparisons with existing literature can provide valuable insights into the prevalence and patterns of maternal complications associated with labor induction. Studies conducted in diverse settings have reported varying rates of maternal complications, influenced by factors such as patient demographics, obstetric practices, and healthcare infrastructure [9, 13]. By synthesizing evidence from multiple studies, clinicians can gain a more nuanced understanding of the risks and benefits associated with labor induction, enabling informed decision-making and individualized patient care [14].

One of the primary neonatal outcomes examined in our study was the Apgar score at five minutes. The Apgar score serves as a valuable tool for assessing the newborn’s overall condition and response to delivery. Our findings indicated that the majority of neonates achieved Apgar scores of seven or above at five minutes, reflecting satisfactory neonatal adaptation to extrauterine life. However, it is important to recognize that low Apgar scores can signal potential neonatal distress or asphyxia, necessitating prompt intervention and neonatal resuscitation.

Meconium-stained liquor was another neonatal outcome of interest in our study. Meconium staining of amniotic fluid can occur due to fetal distress or intrauterine hypoxia and may increase the risk of meconium aspiration syndrome (MAS) and subsequent respiratory complications in neonates. While a proportion of neonates in our study exhibited meconium-stained liquor, the absence of MAS cases suggests that effective management strategies were employed to prevent neonatal respiratory compromise.

Admission to the neonatal ward for further care and management was required for a subset of neonates in our study. Neonatal admission may be warranted for various reasons, including prematurity, respiratory distress, or suspected sepsis. Timely evaluation and supportive care in a neonatal setting are crucial for optimizing neonatal outcomes and reducing the risk of morbidity and mortality in vulnerable newborns [15].

Comparisons with existing literature provide valuable insights into the prevalence and patterns of neonatal outcomes associated with labor induction. Studies conducted across diverse clinical settings have reported varying rates of neonatal complications, influenced by factors such as gestational age, maternal health status, and obstetric interventions [16–18]. By synthesizing evidence from multiple studies, healthcare providers can gain a comprehensive understanding of neonatal outcomes following labor induction, facilitating risk stratification and tailored neonatal care strategies [19, 20].

This study has several notable strengths that underpin its contributions to obstetric research. Firstly, the study’s relevance lies in its focus on a crucial aspect of obstetric care, providing insights into the effects of labor induction on maternal and neonatal health outcomes in Tanzania. Furthermore, the utilization of a retrospective design allowed for the examination of existing medical records, offering a real-world perspective on clinical practices and outcomes.

However, despite its strengths, the study is not without limitations that warrant consideration. One such limitation is the potential for data quality issues due to reliance on medical records, which may introduce inaccuracies or incompleteness and lead to information bias. Another limitation stems from the study’s single-center nature, which may limit the generalizability of findings to other settings and populations. While this limitation cannot be fully eliminated, efforts were made to acknowledge and contextualize the study’s findings within the specific study site and population, thereby providing insights relevant to similar contexts.

## Conclusion

The study at Muhimbili National Hospital in Tanzania found favorable outcomes with labor induction despite a low rate compared to developed countries. Predominant indications included postdate pregnancy and hypertensive disorders, with minimal complications. Most deliveries were spontaneous, and neonatal outcomes were generally positive, although some neonates required additional care. These findings emphasize the need for tailored obstetric care and further research to improve maternal and neonatal health globally.

### Electronic supplementary material

Below is the link to the electronic supplementary material.

Supplementary Material 1

## Data Availability

Availability of Data and Materials: The datasets used and/or analyzed during the current study are available from the corresponding author on reasonable request.
